# Pumpkin seed oil: unveiling its potential in controlling inflammation and pathogenicity during experimental trichinellosis

**DOI:** 10.1186/s12917-024-04241-2

**Published:** 2024-09-20

**Authors:** Sara Salah Abdel-Hakeem, Sulaiman Mohammed Alnasser, Alotaibi Meshal, Mahmoud Abdel-Zaher Abdel-Samiee, Mohamed Salah Eldin Youssef, Shimaa Hamdi Abd Elsadek, Salwa Mahmoud Abd-Elrahman

**Affiliations:** 1 Parasitology Laboratory, Zoology and Entomology Department, Faculty of Science, Assiut University, Assiut, 71526 Egypt; 2 Department of Pharmacology and Toxicology, College of Pharmacy, Qassim University, 52571 Buraydah, Saudi Arabia; 3 College of Pharmacy, University of Hafr Albatin, 39911 Hafr Albatin, Saudi Arabia; 4 Department of Pathology and Clinical Pathology, Faculty of Veterinary Medicine, Assiut University, Assiut, 71526 Egypt; 5 Department of Pathology and clinical pathology, Faculty of Veterinary Medicine, Sphinx University, Assiut University, Assiut, 71526 Egypt; 6 Department of Parasitology, Faculty of Veterinary Medicine, Assiut University, Assiut, 71526 Egypt

**Keywords:** *Trichinella*, Pumpkin seed oil, MMP-9, Anti-inflammatory, Antiparasitic

## Abstract

**Background:**

This study aimed to investigate the antiparasitic and anti-inflammatory potential of pumpkin seed oil in mice infected with *Trichinella spiralis* by demonstrating its impact on MMP-9 expression and pathogenesis during the intestinal and muscular phases.

**Results:**

In this study, 100 mice were divided into five groups: an infected group, a pumpkin seed oil-treated group (1.5 mg/kg BW, administered three times per week), an albendazole-treated group, a native control group, and a pumpkin oil control group. Gas chromatography–mass spectrometry analysis of the pumpkin seed oil revealed a broad spectrum of biologically active compounds. The pumpkin seed oil treatment led to a significant reduction in the parasite burden, with a 75% decrease in adult worms and a 66% decrease in encysted larvae. Additionally, the infected animals treated with pumpkin oil exhibited a marked reduction in intestinal inflammation, characterized by a progressive increase in goblet cells. The number of encysted larvae in the diaphragm and muscle tissues was also significantly decreased. Furthermore, pumpkin seed oil treatment significantly reduced MMP-9 levels in both intestinal and muscular tissues, highlighting its potential to attenuate inflammation.

**Conclusion:**

These findings underscore the effectiveness of pumpkin seed oil as anti-inflammatory and antiparasitic agent.

**Supplementary Information:**

The online version contains supplementary material available at 10.1186/s12917-024-04241-2.

## Introduction

*Trichinella spiralis* is a tiny nematode parasite found in rodents, pigs, and humans. It is a highly prevalent and significant parasite that causes trichinellosis globally [1]. This parasite is commonly used to test the effectiveness of different anthelmintic drugs [2]. Albendazole, a broad-spectrum anthelmintic drug, is the primary medication used to treat trichinellosis [3]. Unfortunately, its effectiveness is limited due to its low water solubility and the high level of resistance exhibited by parasitic stages [4]. Additionally, it has many side effects including neurological symptoms such as headaches, nausea, abdominal pain, fever [5]. Other side effects such as leukopenia, anemia, thrombocytopenia, and pancytopenia, and elevation in liver enzymes were also reported [6]. In neuro-helminth infection, albendazoles may cause focal neurologic deficits due to the destruction of helminthic larvae and cysts in the brain, in addition to meningeal signs and increased intracranial pressure [7]. Besides, it is restricted for pregnant women and children under the age of three years [2], and some are suspected to be carcinogenic [8]. Therefore, it is crucial to find safe and effective anti-Trichinellosis drugs, particularly natural agents with low cost, ecofriendly, and with no adverse effects [9]. Throughout history, natural plant extracts have emerged as powerful alternatives or complementary therapies against parasitic diseases. Pumpkin with oily seeds belong to the Cucurbitaceae family and are used to relieve several conditions, such as fever, bronchitis, and sore chests, as well as a diuretic and tonic. Previous literature demonstrated the therapeutic application of pumpkin oil against benign prostatic hyperplasia [10], cardiovascular health [11], antimicrobial properties [12], diabetes management [13], and against parasitic diseases [14–19]. The detailed physiochemical properties of pumpkin seed oil have been evaluated by our colleagues in the Faculty of Agriculture at Assiut University [20]. Pumpkin seed oil contains bioactive compounds that possess anthelmintic properties and is predominantly composed of four fatty acids: linoleic, oleic, stearic, and palmitic with a relative distribution of 33.1%, 43.8%, 7.8%, and 13.4%, respectively [21, 22].

It contains a specific amino acid that plays a major role in eliminating worms and is mainly concentrated in the seeds of *Cucurbita* species [23, 24].

Several studies have explored the diverse pharmacological effects and parasitological impacts of pumpkin on nematodes [17–19, 25]. This has been found to decrease the number of parasite eggs [26]. The in vitro studies revealed that the alcoholic extract of pumpkin (*Cucurbita pepo*) completely inhibits the mobility of *T. spiralis* and *T. britovi* larvae [27]. Furthermore, Abd Elsadek et al. [28] demonstrated the impact of pumpkin seed oil on the hepatic pathogenicity and inflammatory reactions resulting from *T. spiralis* infection.

Matrix metalloproteinases (MMPs) are endogenous regulators involved in tissue regeneration and inflammation. They play an essential role in granuloma formation during infection by promoting the infiltration of inflammatory cells and degrading extracellular matrix proteins [29]. In terms of parasitic infections, researchers have extensively studied the functions of these proteins in diseases such as malaria, neurocysticercosis, and angiostrongyliasis [30, 31]. In addition, MMP-9 is also known to have the ability to both generate and resolve fibrosis in the liver [28]. Many studies have reported a significant increase in the serum levels of MMPs in mice infected with *T. spiralis* and, to a lesser extent, in mice infected with *T. pseudospiralis* [32]. These results suggested that MMPs, specifically gelatinases, may serve as inflammatory markers [30]. To the best of our knowledge, little information is available regarding the role of pumpkin against *T. spiralis* infection. Therefore, the current study aimed to explore the effect of pumpkin seed oil on the enteral and parenteral phases of murine trichinellosis by reducing the pathogenesis and production of the inflammatory mediator MMP-9.

## Materials and methods

### Ethical standards

This study adhered to both national and international ethical guidelines. The study was approved by the Research Ethical Committee of the Faculty of Veterinary Medicine, Assiut University, Egypt, according to the guidelines of The OIE standards for the use of animals in research (protocol code: 06/2023/0140).

### Materials

Pumpkin seed oil (purity 100%) was purchased from IMTENAN brand for Natural Oils and Herbs, Egypt. The oil was extracted using a cold-pressed method that preserves its natural benefits. It has a dark brown to green to dark red color with a strong nutty aroma. The oil is 100% pure and natural, and it has been certified by ISO, COA, and MSDS.3.5. Albendazole was provided as a suspension (Alzentale) by Egyptian International Pharmaceutical Industries [33].

### Gas chromatography–mass spectrometry (GC–MS) analysis of the pumpkin seed oil

The phytochemical constituents of the pumpkin seed oil were analyzed with GC/MS Agilent 6890 gas chromatograph equipped with an Agilent mass spectrometric detector at Egyptian Pharmacopoeia, using a direct capillary interface and fused silica capillary column PAS-5ms (30m × 0.32 mm × 0.25μm film thickness). The oil under investigation was injected under the following conditions: helium was used as carrier gas at approximately 1.0 ml/min, pulsed split less mode, the solvent delay was 3 min, and the injection size was 1.0 μl. The mass spectrometric detector was operated in electron impact ionization mode with ionization energy of 70 e.v. scanning from m/z 50 to 500. The ion source temperature was 230 multiplier voltage (EM 5 voltage) maintained 1250 v above auto tune. The identification of compounds was achieved by library search on a Wiley 275 L GC/MS database (Thermo Fisher Technology, Waltham, Massachusetts, United States) and using AMDIS software (www.amdis.net), identified by its retention indices (relative to n-alkanes C8.0 – C24.0) and mass spectrum matching to available authentic standards. Wiley spectral library collection and the National Institute of Standards and Technology (NIST) library database curves generated by running GC analysis of representative authentic compounds.

### Parasite strain

The strain of *T. spiralis* isolated from naturally infected pig obtained from El-Bassatine Abattoir, Cairo. In brief, the infected carcasses were skinned, minced, digested in digestive fluid, and then incubated at 37 °C overnight. Larvae were filtered using thieve to remove bones and hair then washed in PBS. The precipitated larvae were washed several times in PBS, the larvae number per ml was counted using light microscope (× 40). BALB/c mice were administered orally with 350 larvae under appropriate conditions and free of pathogens. The infection was maintained via regular passage in the Animal House, Faculty of Veterinary Medicine, Assiut University, Egypt. The larvae were recovered from the carcasses 30 days post-infection [34].

### Experimental design

One hundred BALB/c mice (age 8–12 weeks, weighed 25-30 g) were purchased from the Animal House, Theodore Bilharz Research Institute, Cairo, Egypt. Animals were divided into the main five groups Table 1: the infected untreated group was administered orally with 350 *T. spirallis* larvae, the pumpkin-treated group was administered orally with 1.5 ml/kg BW pumpkin seed oil three times/ week [28, 35], albendazole-treated group was administered orally with 50 mg/kg BW [36]. The negative control and pumpkin control groups were also represented. The animals of all groups were euthanized at the end of the 7 day post infection (dpi) and 54 dpi for enteral and parenteral phase, respectively and anesthetized with intraperitoneal injection of sodium thiopental (100 mg/kg) [37, 38].

**Table 1:** Experimental design showing the experimental groups and subgroups, dose of infection, and treatment

Animal group		Subgroups
**GI (10 mice)**	Control non-infected, non-treated group	––
**GII (15 mice)**	Pumpkin non-infected group	––
**GIII (15 mice)**	Infected non-treated group (orally administrated with 350 *T. spirallis* larvae)	––
**GIV (30 mice)** **Pumpkin- treated group**	Infected mice were administered orally with 1.5 ml/kg BW pumpkin seed oil three times/ week according to Abd ELsadek et al*.,* 2023 and Elhamalawy, 2018.	• IVa (enteral phase): 15 infected mice were treated with pumpkin seed oil from 2nd till 7th dpi
		• IVc (parenteral phase): 15 infected mice were treated with pumpkin seed oil from 7th till 54th dpi
**GV (30 mice)** **Albendazole-treated group**	Each infected mouse was administered orally with Albendazole, as a reference drug (50 mg/kg BW) according to Abdel-Hakeem et al*.,* 2024 and El-Hamed et al., 2022.	• Va (enteral phase): 15 infected mice were treated with albendazole from 3rd for three successive days
		• Vb (parenteral phase): 15 infected mice were treated with albendazole from 31th for seven successive days

### Parasitological parameters

#### Evaluation of the adult worm burden in intestine

Mice in each group were euthanized, and the small intestines were removed, washed multiple times, and then gently scraped to expose the mucosa. The washed intestines were placed in phosphate buffered saline (PBS) and incubated for 4 h at 37°C to allow the worms to migrate out of the tissue and gather in a petri dish. Adult worms were examined, and the mean number was estimated under a stereomicroscope at a magnification of × 10 [33].

#### Evaluation of the larval burden in muscle

Briefly, 54 dpi mice were sacrificed, the skin of each mouse was removed, rectus abdominis muscles were minced, and digested using artificial digested fluid containing 1% pepsin (1:10,000) and 1% HCl in 200 mL distilled water. The mean number of encysted larvae was determined according to Goettstein et al. [3]. The larvae were counted using a stereomicroscope at a magnification of 40 × . The total number of larvae in the carcass was estimated by multiplying the number of larvae in 50 µl by 8000 (dilution factor).

The efficacy of treatment was counted according to Attia et al*.* [33]

$$\text {Efficacy of treatment}\ (\%)=100 \times (\text {Mean No. recovered in control - Mean number recovered in the treated group}/\text{Mean No. recovered in the control})$$

#### Histopathology

The intestinal and muscle tissues were separated and preserved in a fixative (formalin-alcohol solution). After 48 h, the specimens were washed several times with 70% ethanol and serially dehydrated in increasing alcohol concentrations. The specimens were cleared in xylene three times/45 min each, impregnated, and embedded in paraplast. Transverse sections were cut at 4–5 µm and stained with hematoxylin and eosin [39, 40].

#### Immunohistochemistry and scoring of MMP-9

For immunohistochemical staining, paraffin-embedded tissues were cut at 3–4 µm on coated slides. All reagents used in the study were from the Dako EnVision™ FLEX system. Citric buffer (50 ×) at a low pH 6.1 (Code DM829) was used as the Antigen Retrieval technique. Peroxidase Blocking Reagent (Code SM801) was applied and incubated at room temperature for 5–10 min. A ready-to-use Tinto prediluted rabbit monoclonal antibody against MMP-9 (Catalog No. BSB 2538; Bio SB clone EP127, USA) was used according to the manufacturer's protocol with a tonsil tissue as a control (Supplementary 1). Horseradish peroxidase secondary antibody (Code SM802) was applied for 20 min at room temperature. DAB solution (Code DM827) was applied for 5–10 min to visualize the bound antibody, and the sections were stained with counterstain with haematoxylin and mounted using Dibutyl Phthalate Polystyrene Xylene. For scoring the immunostaining, three slides from three different animals in each group were examined under a light microscope (OPTICA, Italy) at a fixed high-power field of × 400 magnification. The number of positive cells was counted using ImageJ software, and the mean number of immunopositive cells was expressed [39, 40].

#### Serum level of MMP-9 using enzyme-linked immunosorbent assay (ELISA)

MMP-9 levels were assessed in all groups during the enteral phase on the 7th dpi and the parenteral phase on the 54th dpi using a commercially available quantitative sandwich immunoassay kit (Cat. No. E0321Ra, BT LAB, Shanghai, China). Briefly, 40 µL of serum sample (three replicated/each tested group) was added to a 96-well plate that had been coated with the Rat MMP-9 antibody. Then, 50 µL of streptavidin-HRP was added and mixed thoroughly. The mixture was incubated at 37°C for 60 min and washed with buffer five times/ per minute. Subsequently, equal volumes (50 µL) of substrate solutions A and B were added, and the plate was further incubated for 10 min at 37°C in dark. The stop solution (50 µL) was added, and MMP-9 was determined at an optical density of 450 nm. The standard values ranged from 0.375 to 6 ng/mL. The intra-assay coefficient of variation was 4% and the inter-assay coefficient of variation was 6.1%.

### Data analysis

SPSS software (version 20) and Microsoft Excel sheet (version 2016) were used in analysis, and the data were expressed as mean ± standard deviation. The two-tailed unpaired data was used to evaluate the difference in MMP-9 level. We considered differences significant when *P* was less than or equal to 0.05.

## Results

### GC–MS analysis

**Fig. 1:**
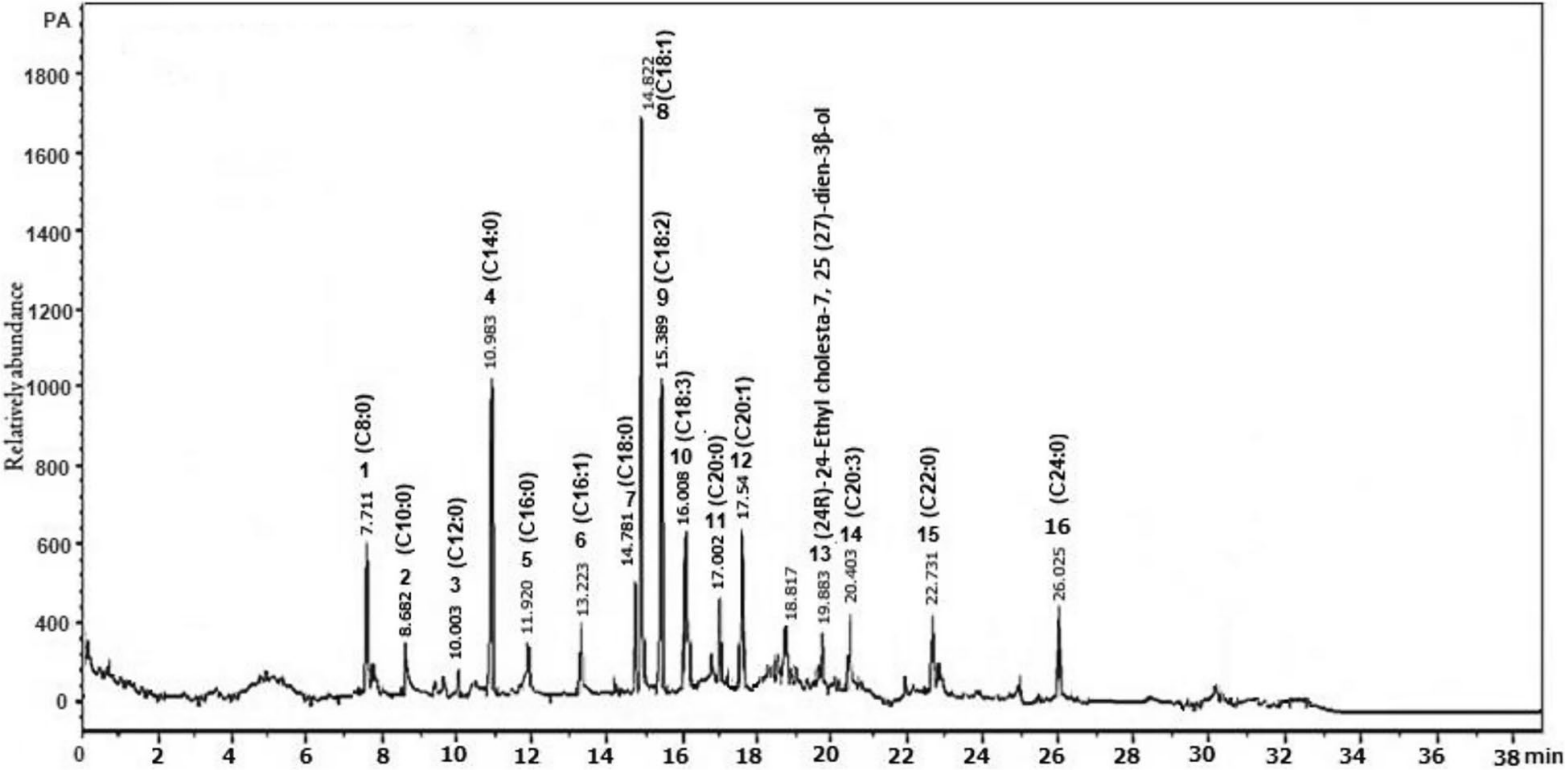
GC-Mass histogram shows the main sixteen phytochemical constituents in the pumpkin seed oil used in the current evaluation

GC–MS analysis of pumpkin seed oil indicated the presence of active sixteen phytochemical compounds (Fig. 1) with highly biological activities as shown in Table 2. The phytochemical constituents of the oil are mainly mono- and poly- saturated and unsaturated fatty acids, esters, carboxylic acid, and alkenes (Fig. 1). Molecular weight, chemical formula, chemical structure, retention time (RT), concentration (peak area %), and detailed biological activities of the detected bioactive compounds are listed in Table 2.

**Table 2:** GC–MS spectral analysis of phytochemical compounds identified in pumpkin seed oil

N	Compound	Molecular weight	Nature	Chemical formula	Chemical structure	Area %	RT	Biological activities
1	Caprylic acid	144.211	Ester	C_8_H_16_O_2_		6.766	7.711	Antioxidant and antibacterial [41]
2	Capric	172.268	Saturated fatty acid	C_10_H_20_O_2_		2.461	8.682	Antibacterial [42]
3	Lauric	200.322	Saturated fatty acid	C_12_H_24_O_2_		0.361	10.003	Antibacterial [43] and anti-inflammatory [44]
4	Myristic	228.376	Saturated fatty acid	C_14_H_28_O_2_		13.421	10.983	Antiviral, antifungal, and anticancer [45]
5	Palmitic	256.430	Saturated fatty acid	C_16_H_32_O_2_		2.154	11.920	Anti-inflammatory and antioxidant [46]
6	Palmitoleic	254.41	Mono unsaturated fatty acid	C_16_H_30_O_2_		2.602	13.223	Anti-inflammatory [47] and antibacterial [48]
7	Stearic	284.484	Saturated fatty acid	C_18_H_36_O_2_		3.141	14.781	Anti-inflammatory and antioxidant [49]
8	Oleic	282.468	Mono unsaturated omega-9 fatty acid	C_18_H_34_O_2_		25.273	14.822	Antioxidant [50], anti-inflammatory and anticancer [51], antiprotozoal, molluscicidal, and insecticidal [52]
9	Linoleic	280.452	alkane	C_18_H_32_O_2_		14.078	15.389	Anti-inflammatory and antioxidant [53]
10	Linolenic	278.4	triglyceride esters	C_18_H_30_O_2_		8.413	16.008	Anti-inflammatory, anticancer, and immunomodulatory [54]
11	Eicosenoic	310.5	Mono unsaturated omega-9 fatty acid	C_20_H_38_O_2_		2.807	17.002	Anti-inflammatory, anticancer, and antibacterial [55]
12	Arachidic	304.474	Poly-unsaturated omega-6 fatty acid	C_20_H_32_O_2_		6.011	17.540	Anti-inflammatory, immunomodulatory, and anticancer [56], antibacterial [57]
13	(24R)-24-Ethyl cholesta-7, 25 (27)-dien-3β-ol	386.7	alkaloid	C_27_H_46_O		2.388	19.883	Antioxidant, anti-inflammatory, antimicrobial, antidiabetic, and anticancer activity [58]
14	Eicosatrienoic	306.4828	carboxylic acid	C_20_H_34_O_2_		3.010	20.403	Anti-inflammatory, immunostimulant, and anticancer [59]
15	Behenic	340.592	carboxylic acid	C_22_H_44_O_2_		3.242	22.731	Antibacterial and immunostimulant [60]
16	Lignoceric	368.63	Saturated fatty acid	C_24_H_48_O_2_		3.871	26.025	Antitumor, antivirus, antioxidative, antibacterial, anti-UV activities [61]

### Effectiveness of pumpkin seed oil on reduction the mean number of the adult worms and encysted larvae

**Fig. 2:**
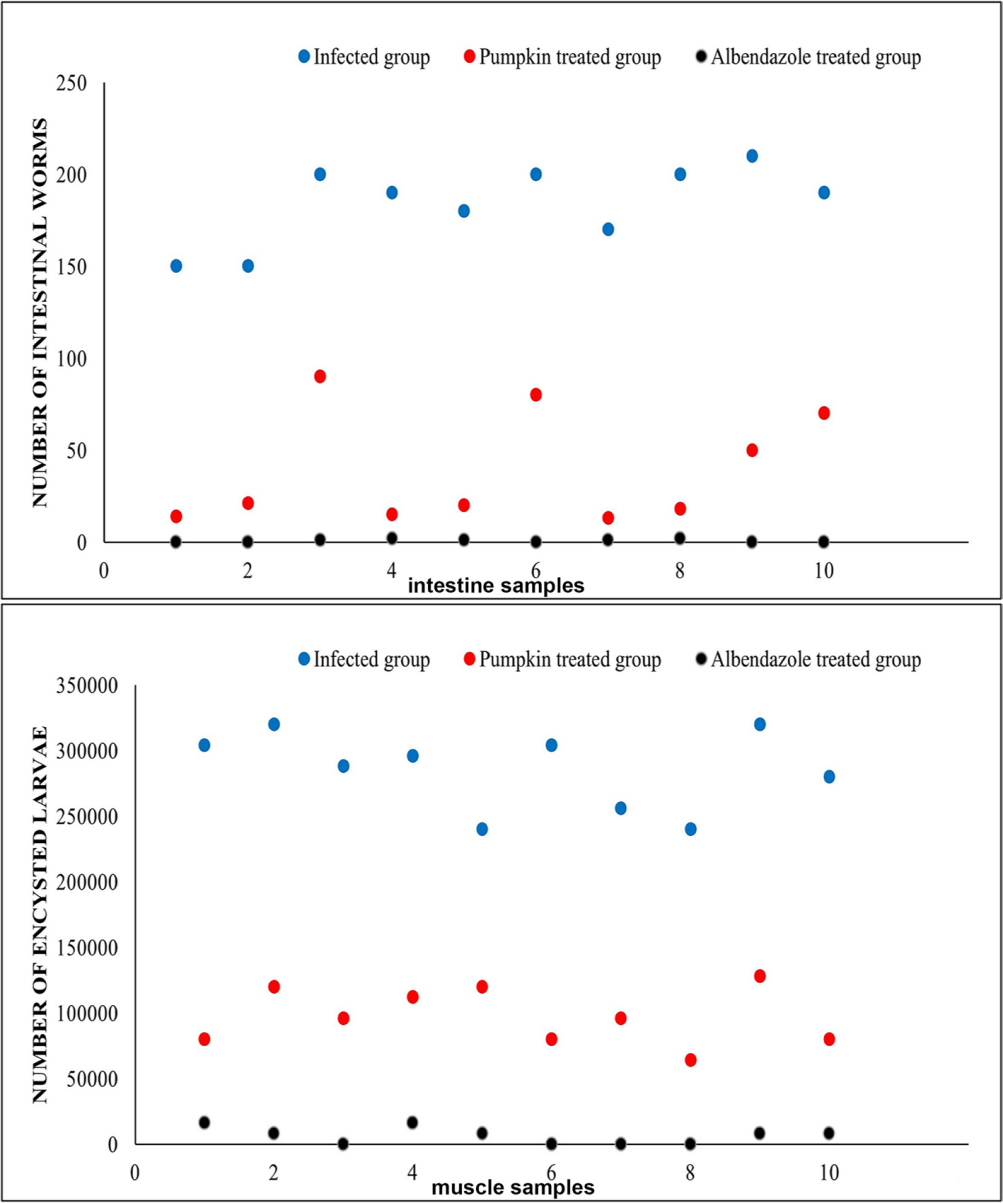
Dot plot shows a marked reduction in mean number of the adult worms (**a**) and encysted larvae (**b**) in the pumpkin and albendazole-treated groups

The pumpkin-treated group showed a significant reduction in the mean number of adult worms (39.1 ± 28.9) and encysted larvae (102,000 ± 27,332.3) with a reduction percentage 78.75% and 64.18%, respectively compared to the infected untreated group (Fig. 2). The albendazole-treated group showed a highly significant reduction (*P* < 0.0001) in the percentage of adult worms and encysted larvae 99.8% and 98.2%, respectively compared to the infected untreated group.

### Histopathological findings

**Fig. 3:**
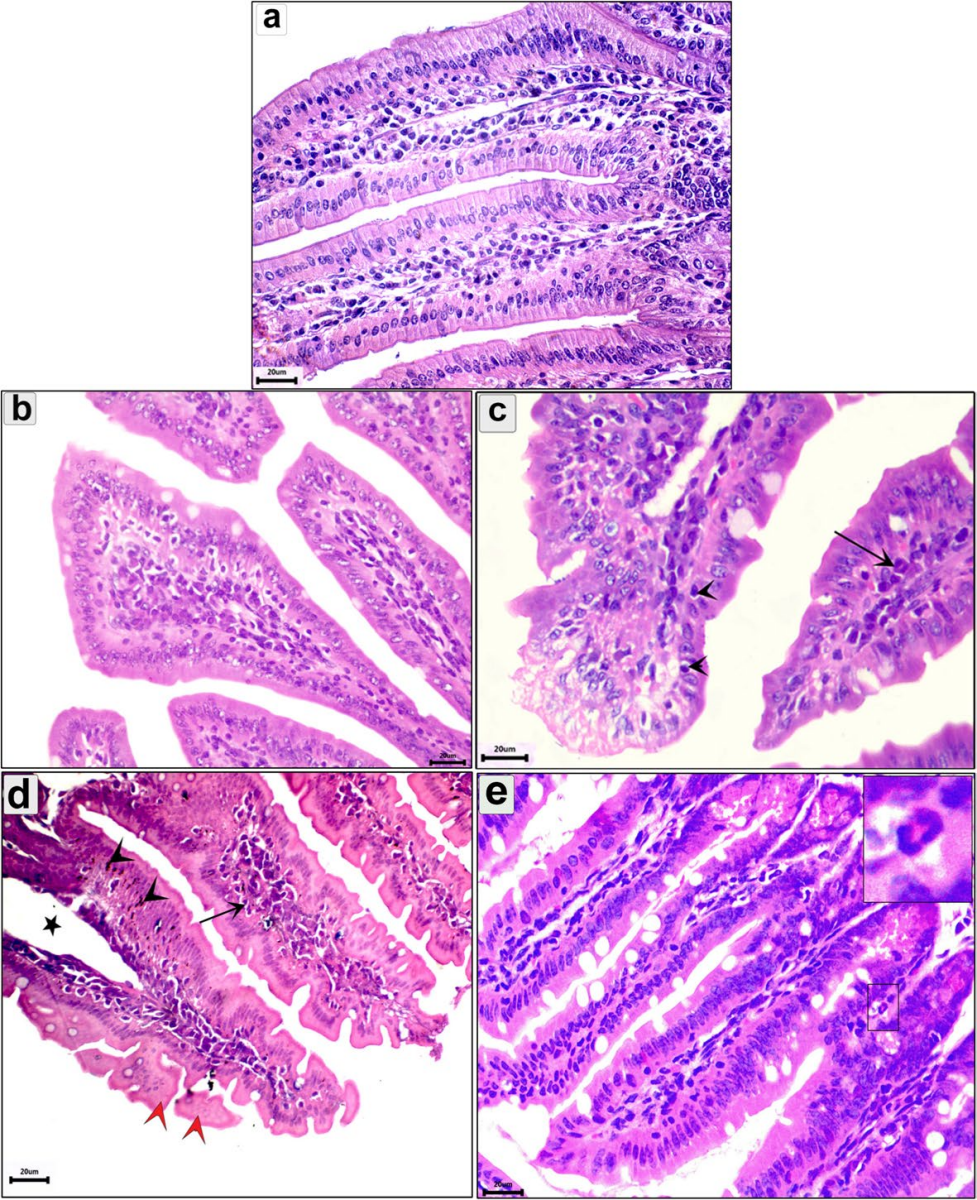
Photomicrograph of the intestine in the different groups in the enteral phase showing: **a** Normal structure of the intestinal mucosa; **b** uninfected pumpkin-treated intestine showing normal villi with healthy enterocytes; **c** Transverse section (T.s) in the intestine of the infected untreated group showing the parasitic enteritis characterized by shedding of the epithelium high inflammatory reaction in the core of the villi (arrow) and mononuclear cells between enterocytes (arrowhead); **d** T.s in the intestine of the pumpkin-treated group showing less normal intestinal mucosa, hyperplasia in some enterocytes (arrowhead), desquamation of the epithelium in tips of villi (red arrowhead), and mild inflammatory reaction in the core of villi (arrow); **e** T.s in the intestine of the albendazole-treated group showing normal intestinal villi with high number of goblet cells and eosinophils infiltration (small box). Stain H&E (400 ×)

**Fig. 4:**
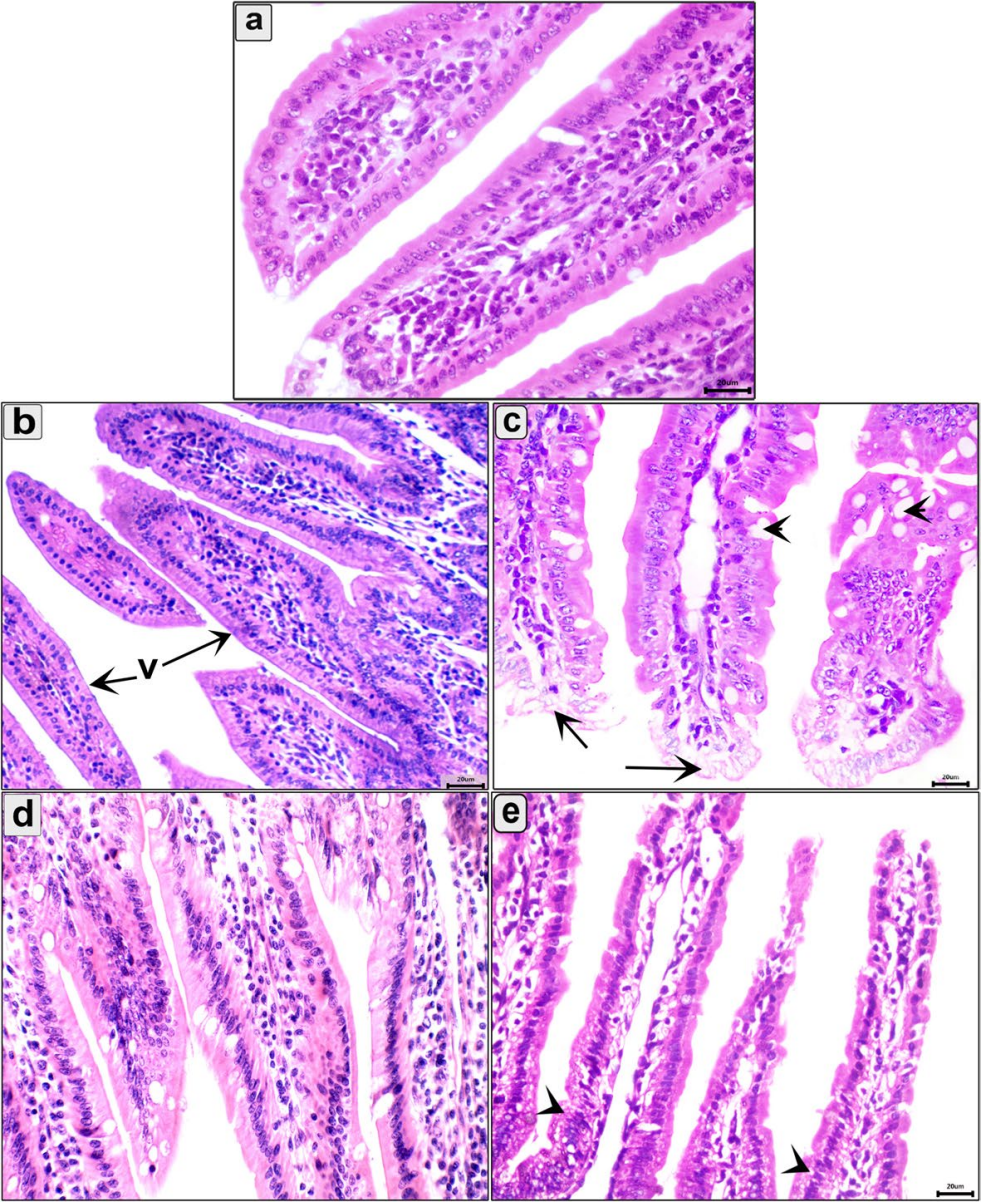
Photomicrograph of the intestine in the different groups in the parenteral phase showing: **a** Normal structure of the intestinal mucosa; **b** T.s in the intestine of uninfected pumpkin-treated showing normal villi (v) with healthy enterocytes; **c** T.s in the intestine of infected untreated group showing vacuolar degeneration in the enterocytes at the tips of villi (arroe) and increase the number of goblet cell (arrowhead); **d** T.s in the intestine of pumpkin-treated group showing normal architecture of intestinal mucosa; **e** T.s in the intestine of albendazole-treated group showing necrobiotic changes and vacuolar degeneration of enterocytes (arrowhead). Stain H&E (400 ×)

Histopathological alteration of the intestine, diaphragm, and muscle were evaluated in all groups. Compared to the normal intestine (Figs. 3a and 4a), the uninfected pumpkin-treated group showed infiltration of mononuclear cells in the intestine (Figs. 3b and 4b). In the enteral phase, the infected-untreated group showed signs of acute catarrhal inflammation, including desquamated epithelium and necrobiotic changes in the enterocytes (Fig. 3c). Furthermore, there were mucosal inflammatory cells, predominantly eosinophils and lymphocytes (Fig. 3c). In the pumpkin-treated group, the intestine showed normal structure with a significant reduction in the enteritis, mild inflammatory reaction in the core of villi, and desquamation of the villar epithelium (Fig. 3d). The intestine in the albendazole-treated group showed normal architecture with inflammatory cell reactions in the core of the villi (Fig. 3e). As shown in Fig. (3c–e), there was a progressively increased number of goblet cells during infection and treatment. In the parenteral phase, the intestine in the infected-untreated group showed milder lesions than in the enteral phase. The intestine showed sloughing of the epithelium in the tips of the villi with a normal number of goblet cells (Fig. 4c). The pumpkin and albendazole-treated groups showed normal intestine structure with a slight inflammatory reaction (Fig. 4d and e).

**Fig. 5:**
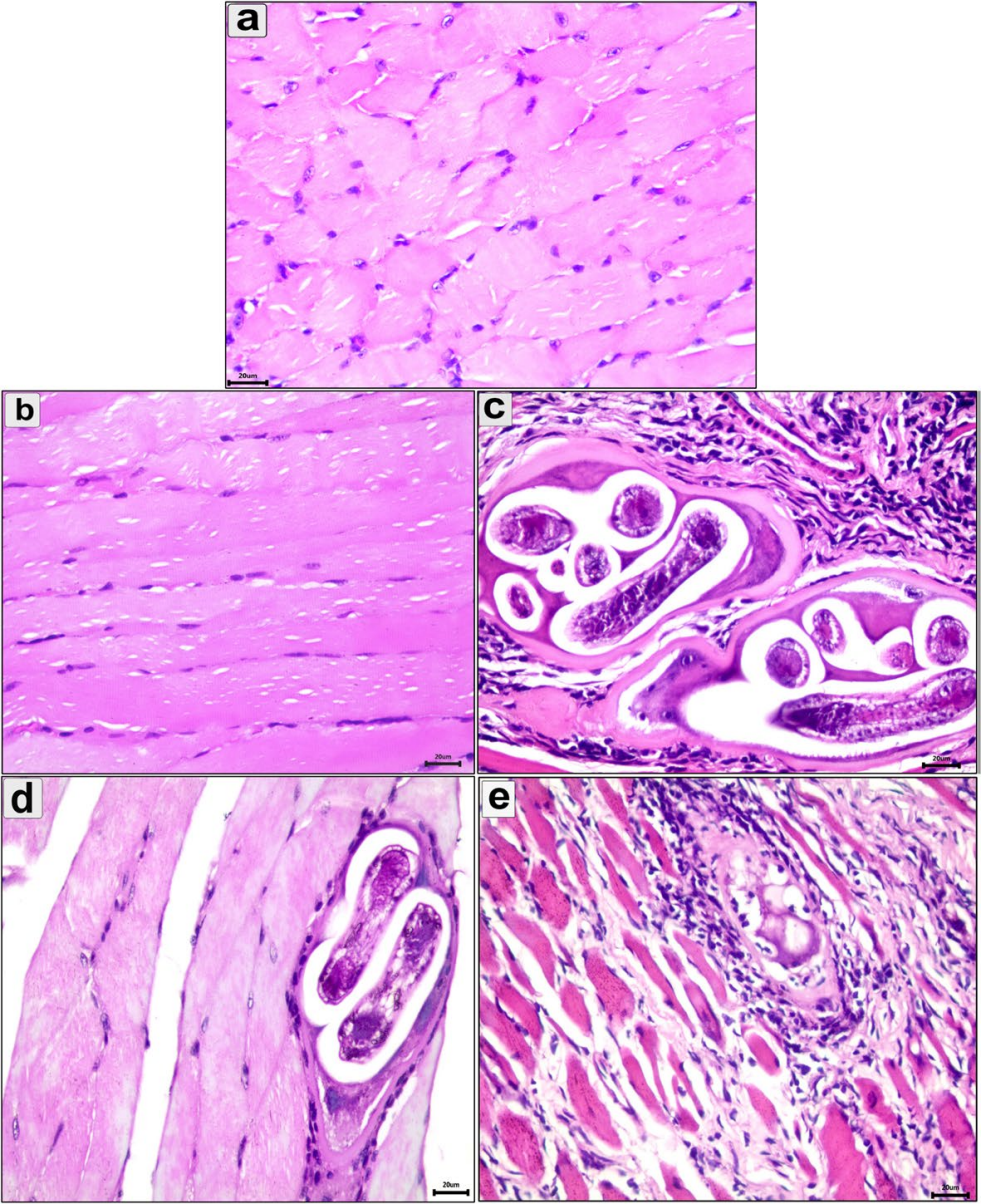
Photomicrograph of the diaphragm in the different groups showing: **a** Normal diaphragm consists of groups of striated muscle fibers; **b** The diaphragm of uninfected pumpkin-treated group showing normal muscle fibers; **c** The diaphragm of infected untreated group showing multiple well-developed cysts containing many larvae surrounded by connective tissue capsule; **d** The diaphragm of pumpkin-treated group showing marked reduction in the number and size of encysted larvae with normal architecture of the muscle fibers; **e** The diaphragm of albendazole-treated group showing degeneration larvae and muscle fibers with marked inflammatory reaction. Stain H&E (400 ×)

**Fig. 6:**
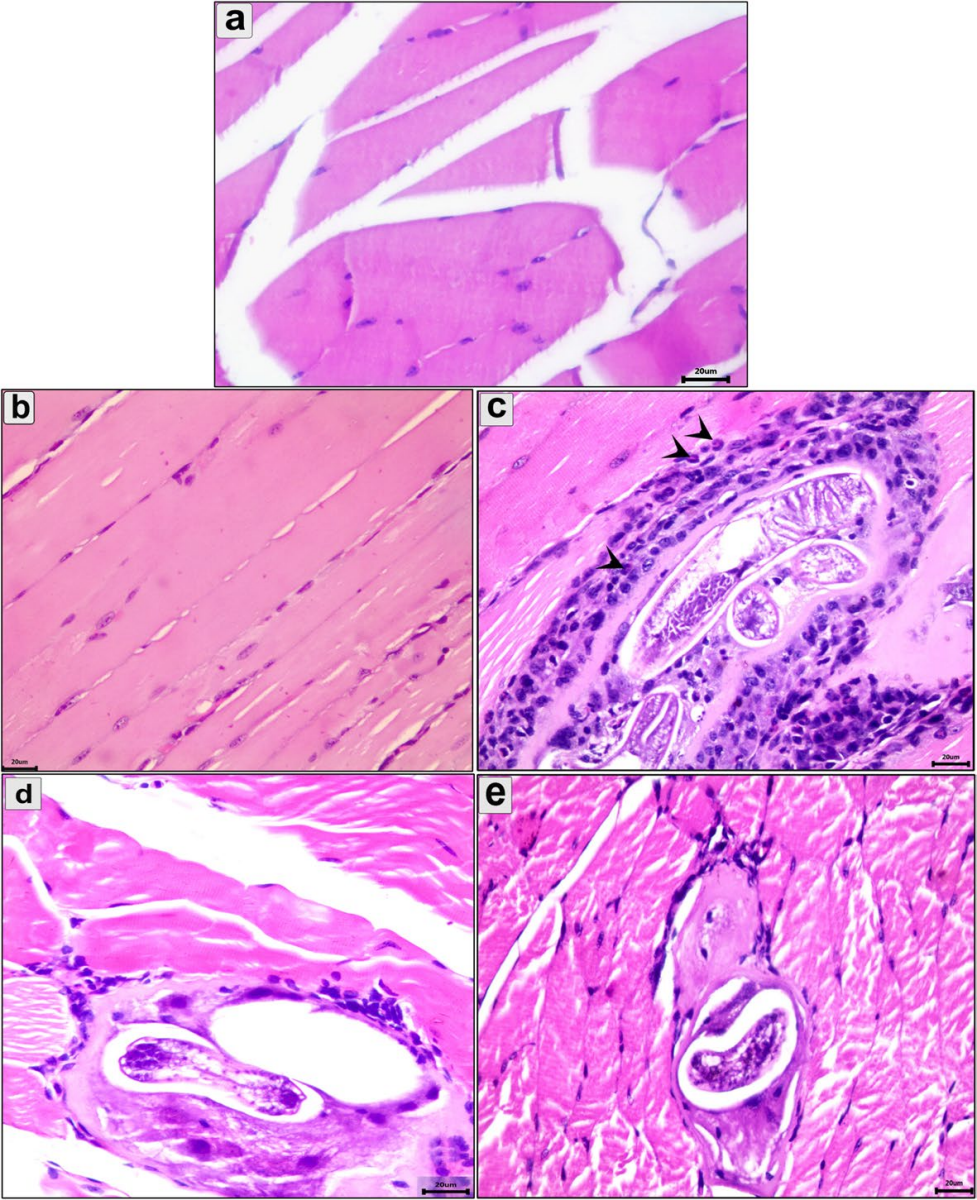
Photomicrograph of the muscle in the different groups showing: **a** Normal structure of muscle consists of bands of muscle fibers; **b** Muscle section of the uninfected pumpkin-treated group showing health muscle fibers with mild inflammatory reaction; **c** Muscle section of the infected untreated group showing multiple larval cysts surrounded by severe inflammatory cells, particularly eosinophils (arrowhead); **d** Muscle section of the pumpkin-treated group showing a degenerative encysted larvae with mild inflammatory reaction; **e** Muscle section of the albendazole-treated group showing minute encysted larvae with mild degeneration in the muscle fibers. Stain H&E (400 ×)

In the diaphragm and muscle, the control (Figs. 5a and 6a) and the uninfected pumpkin-treated group (Figs. 5b and 6b) showed healthy muscle bundles with flat nucleuses. Multiple cysts containing developed and viable larvae were observed in the infected-untreated group (Figs. 5c and 6c). These cysts were surrounded by a connective tissue capsule and had an inflammatory cell reaction from fibroblast and eosinophil cells (Figs. 5c and 6c). This caused severe parasitic myositis, which was represented by coagulative necrosis, vacuolation, and degeneration of muscle fibers. The pumpkin and albendazole-treated groups showed a significant reduction in the number of encysted larvae in the diaphragm (Fig. 5d and e) and muscle (Fig. 6d and e), which were completely degenerated. An extensive inflammatory cell reaction was observed between muscle fiber and the surrounding encysted larvae.

### Downregulation in the expression of MMP-9 in the intestine, diaphragm, and muscle and accompanied with reduction of MMP-9 level

**Fig. 7:**
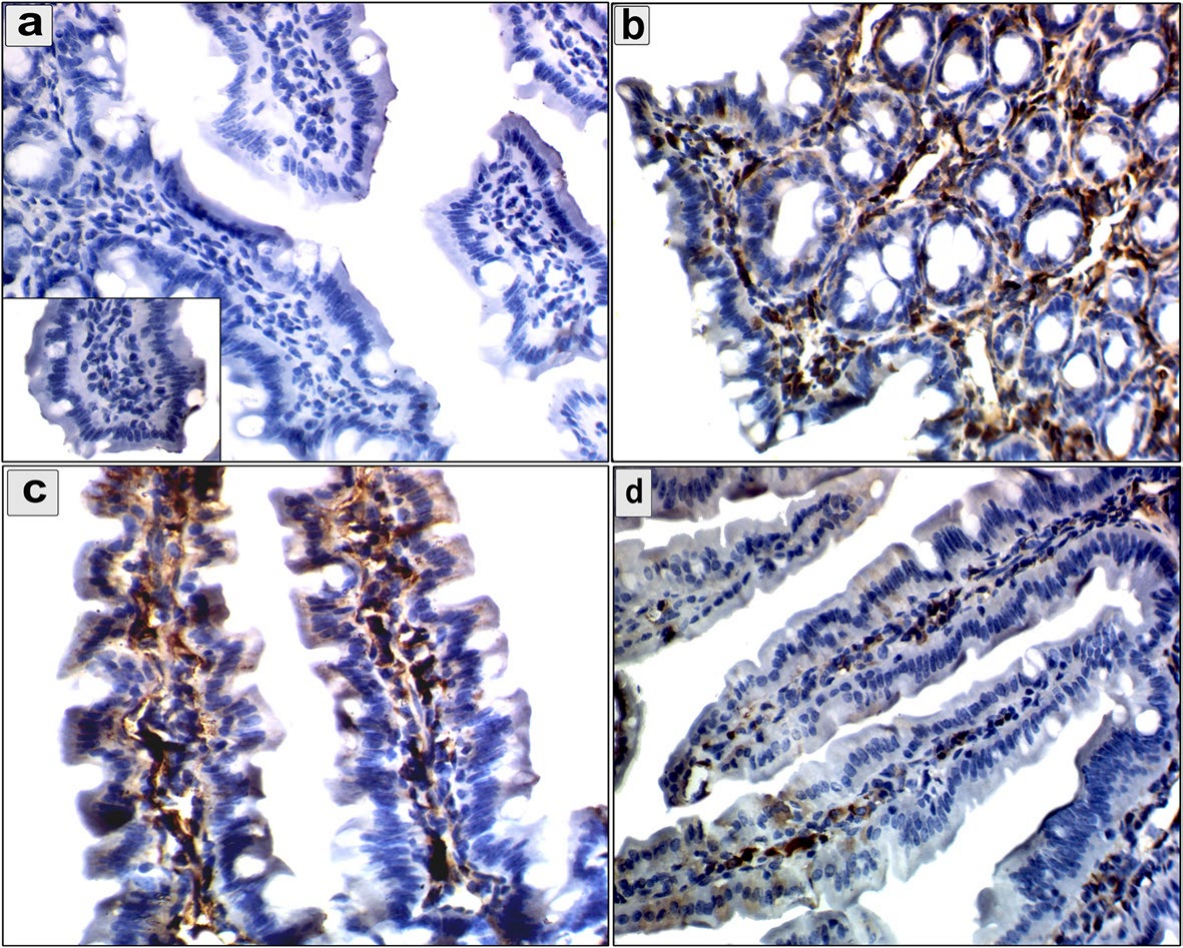
An immunohistochemical staining of MMP-9 the intestine demonstrates the anti-inflammatory effects of pumpkin oil and albendazole (**a**) The negative reaction in the uninfected pumpkin-treated group (negative control in the small box); **b** Severe reaction of MMP-9 positive cells in the infected untreated group were distributed in the mucosa and extending to the submucosa; **c** Marked downregulation in the MMP-9 expression in the pumpkin-treated group and the albendazole-treated group (**d**)

**Fig. 8:**
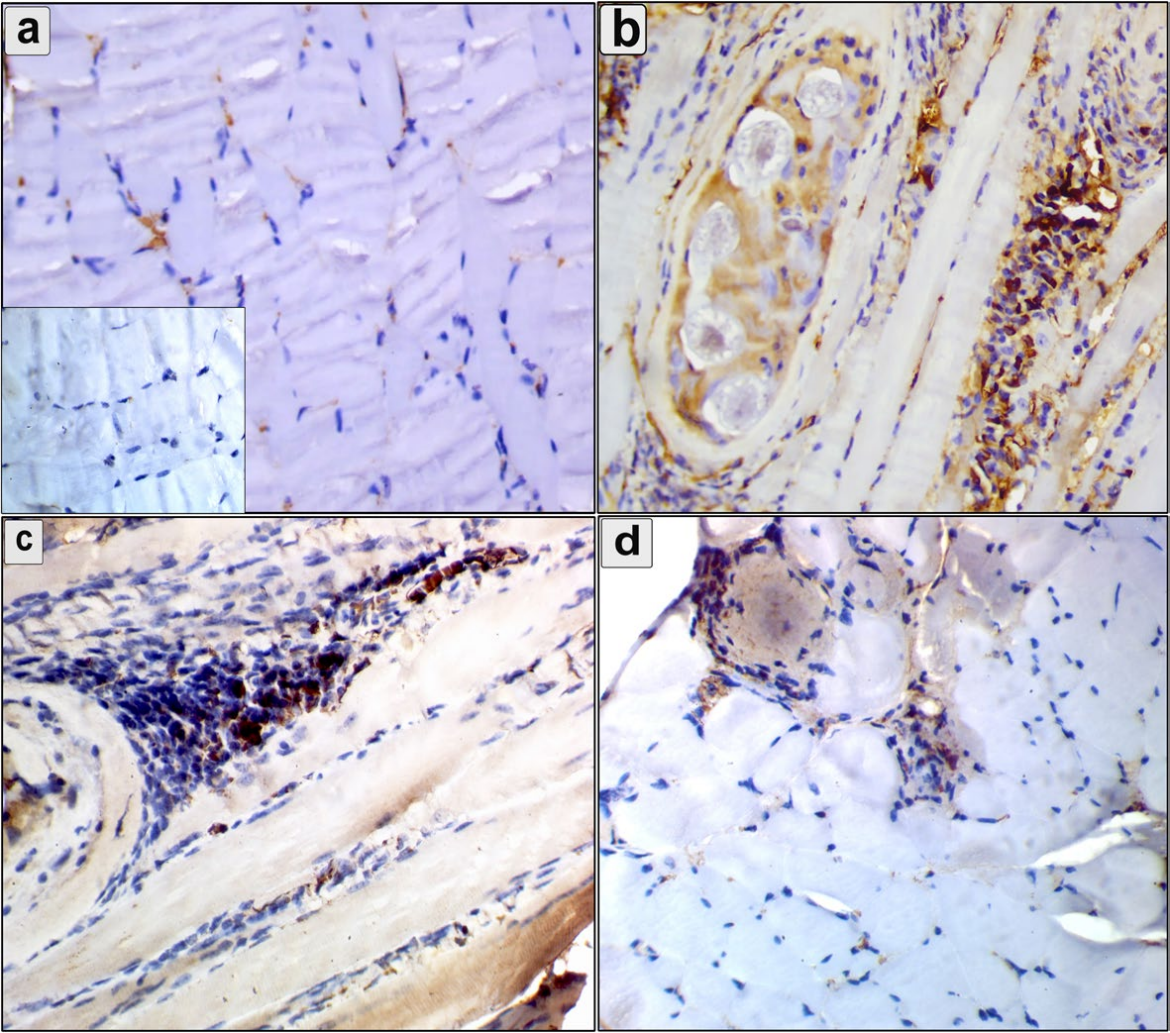
An immunohistochemical staining of MMP-9 in the diaphragm demonstrates the anti-inflammatory effects of pumpkin seed oil and albendazole (**a**) The negative reaction in the uninfected pumpkin-treated group (negative control in the small box); **b** Cytoplasmic reaction of MMP-9 positive cells were distributed around encysted larvae and between muscle bands in the infected untreated group; **c** Marked downregulation in the expression of MMP-9 around the encysted larvae in the pumpkin-treated group and the albendazole-treated group (**d**)

**Fig. 9:**
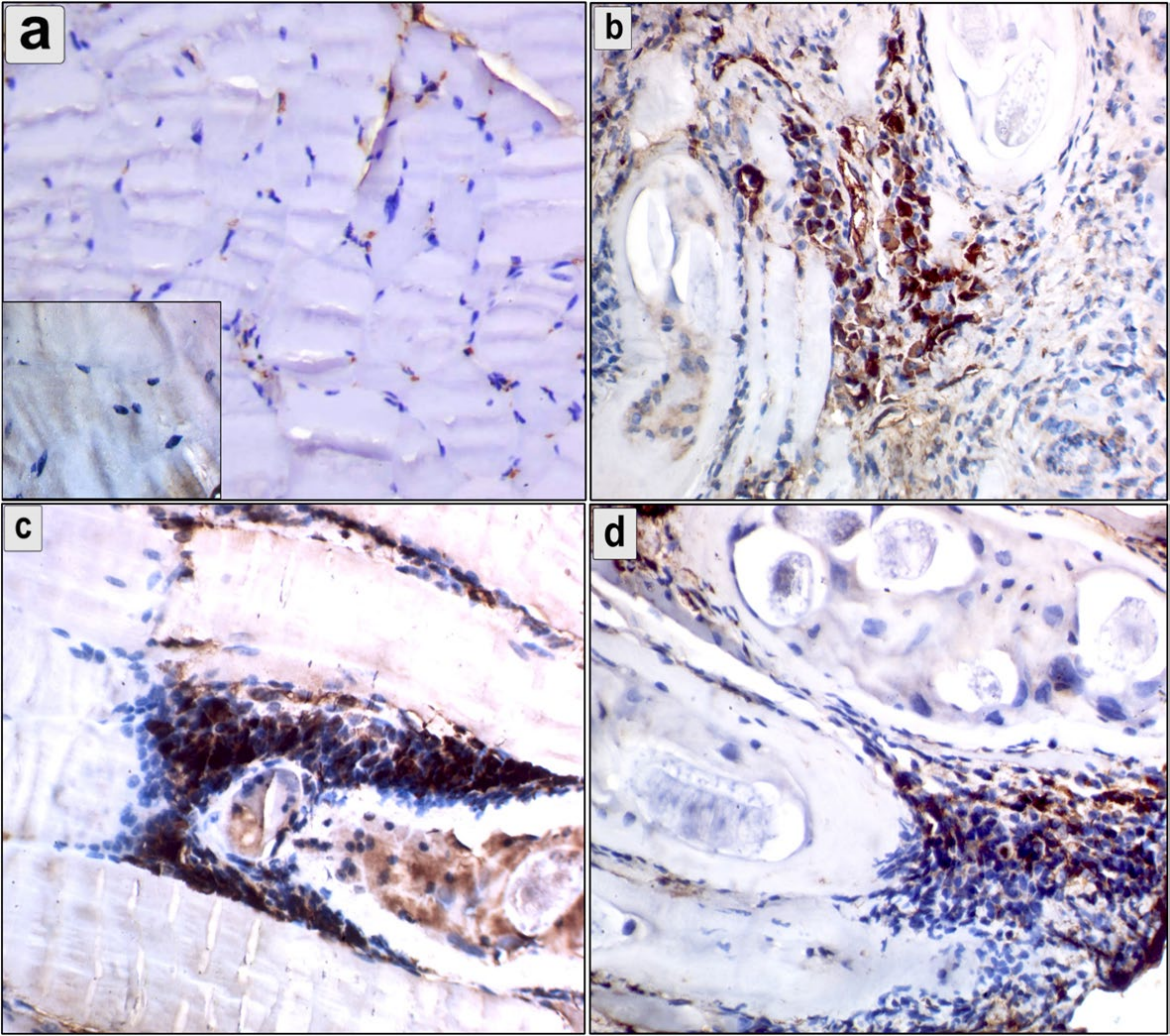
An immunohistochemical staining of MMP-9 in the muscle demonstrates the anti-inflammatory effects of pumpkin and albendazole. **a** The negative reaction in the uninfected pumpkin-treated group (negative control in the small box); **b** Cytoplasmic reaction of MMP-9 positive cells in the infected untreated group were distributed between muscle fibers and around the encysted larvae; **c** non-significant reduction in the MMP-9 expression was observed around the encysted larvae and between muscle bands in the pumpkin-treated group and the albendazole-treated group (**d**)

**Fig. 10:**
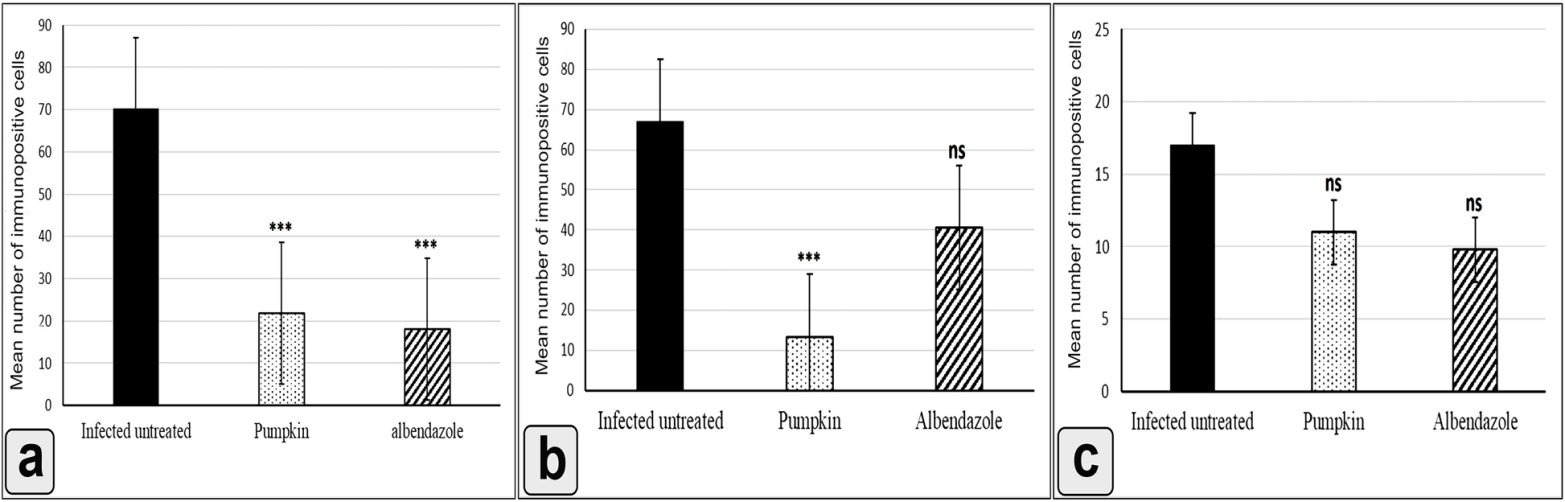
Histogram showing the mean number of MMP-9 positive cells in the intestine (**a**), diaphragm (**b**), and muscle (**c**) of the different groups. Differences were highly significant at ***P ≤ 0.0001, ns, non-significant

The immunohistochemical analysis showed cytoplasmic reaction of MMP-9 in the intestine, diaphragm, and muscles (Figs. 7, 8 and 9). The mean number of immunopositively cells in the intestine, diaphragm, and muscle was assessed (Fig. 10). The pumpkin and albendazole-treated groups exhibited a marked significant reduction (*P* = 0.0007) in the expression of MMP-9 in the intestine (Fig. 7c and d) compared to the infected-untreated group (Fig. 7b). Additionally, the pumpkin-treated group (Fig. 8c) showed a highly significant reduction in the expression of MMP-9 in the diaphragm (*P* < 0.05), whereas a non-significant expression in the albendazole-treated group (Fig. 8d). Contrarily, both the pumpkin and albendazole-treated groups showed a nonsignificant reduction (*P* = 0.262) in the expression of MMP-9 in the muscle (Fig. 9c and d) compared to the infected-untreated group (Fig. 9b).

**Fig. 11:**
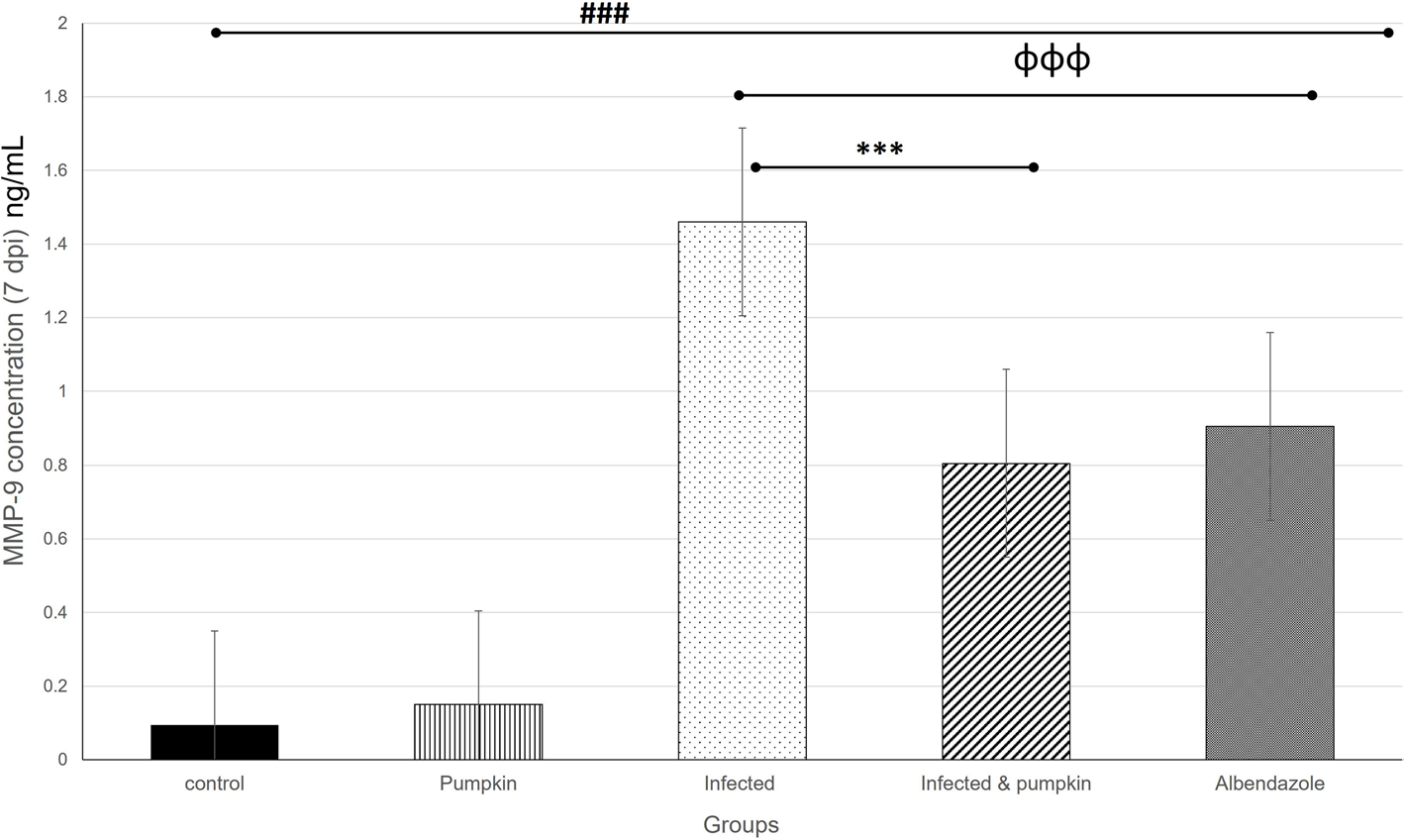
Histogram showing the quantitative level of MMP-9 in the enteral phase of the different groups. A highly significant increase in the level of MMP-9 was observed between uninfected and infected groups. A significant reduction in the treated groups compared to the infected untreated group. Differences were highly significant at *P* ≤ 0.0001

**Fig. 12:**
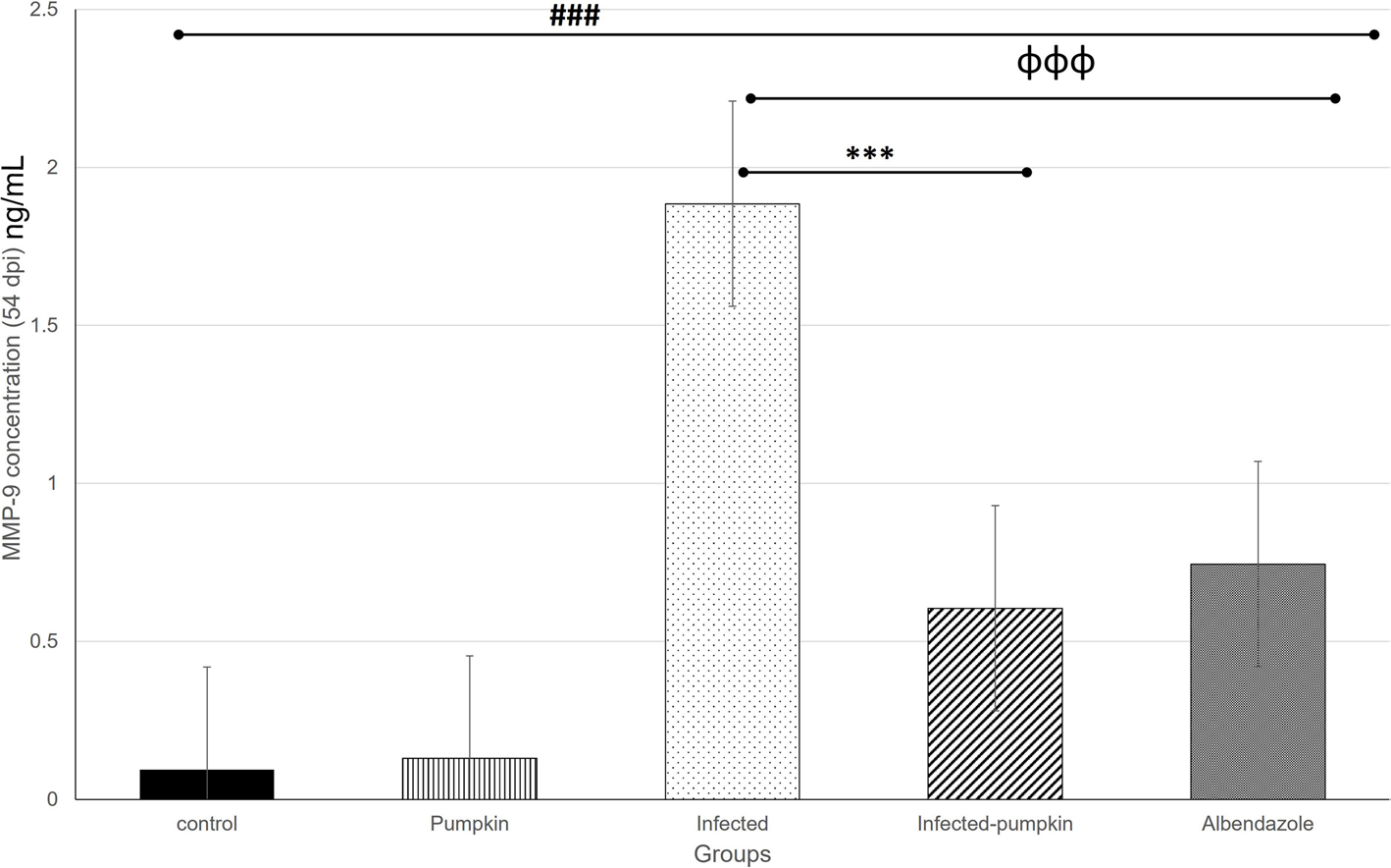
Histogram showing the quantitative level of MMP-9 in the parenteral phase of the different groups. A highly significant increase in the level of MMP-9 was observed between uninfected and infected groups. A significant reduction in the treated groups compared to the infected untreated group. Differences were highly significant at *P* ≤ 0.0001

The level of MMP-9 in the different groups was determined using the ELISA technique in the enteral (7dpi) and parenteral phase (54dpi) of infection. Our results revealed a significant decrease (*P* = 0.0001) in the level of MMP-9 between the infected and treated groups in the enteral (Fig. 11) and parenteral (Fig. 12) phase. The pumpkin and albendazole-treated groups demonstrated a statistically significant reduction in the level of MMP-9 compared to the infected-untreated group.

## Discussion

Pumpkin seeds, as a natural supplement, can influence the hosts’ ability to cope with the negative impact resulting from parasitism and improve the host immunity. In this study, we explore the anthelminthic impact of pumpkin seed oil on the enteral and parenteral phases of *T. spiralis* infection, demonstrating its role in diminishing the inflammatory process. Our GC-Mass analysis shows that the pumpkin seed oil contains mono and poly saturated and unsaturated fatty acids which have a marked biological activity as antibacterial, antiparasitic, anti-inflammatory, and antioxidant. As antiparasitic agent, our results show successfully reduced the adult worm burden and muscle larval load of *T. spiralis* by 75% and 66%, respectively in the pumpkin-treated group. Grzybek et al. [19] previously studied the nematocidal activity of pumpkin extract, reporting that it reduced the egg hatching, larval development, and motility of adult worms of *Heligmosoides bakeri *in vitro. The in vitro impact of pumpkin seed oil against nematodes was also reported against larval stages of *Haemonchus contortus* [62], and *Ascaridia galli* [17]. *In an vivo* study, the extract of pumpkin showed the vigorous antiparasitic effect at high doses [19]. Maciel et al*.*[63] demonstrated that active substances such as nitrogen-containing chemicals in pumpkins have larvicidal and ovicidal effects, which may have inhibited larval development. Furthermore, anthelminthic impact of pumpkin seeds might be attributed to the secondary metabolites which correlated with get rid of the parasites from the host and weakening the attachment between the parasite and the intestinal mucosa [14]. In our experiment, the uninfected mice orally administered with the pumpkin seed oil exhibited a negative effect on the histological architecture of the intestine and muscle tissues with mild inflammatory reaction in the intestinal mucosa and around encysted larvae. This could be attributed to the activation of innate immune reaction. However, the pumpkin seeds possess a proteolytic effect and can damage the tegument, including the basal membrane [64]. Previous histological investigations showed a negative effect of pumpkin seeds on the general health of animal models such as rat and swine [65]. Our published data showed that pumpkin seed oil could diminish the liver damage and reduce the oxidative stress, which occasionally accompanies *T. spiralis* infection, potentially aiding in the recovery mechanism [28]. This could be attributed to the trace minerals such as zinc in pumpkin seeds, acting as an antioxidant having the ability to neutralize free radical generation and/or directly bind to the iron or copper binding sites of lipids, proteins, and DNA molecules [66].

The intestine, diaphragm, and muscle were the main organs affected by *T. spiralis* infection. In the current study, the intestine of the infected-untreated group showed marked parasite enteritis and inflammatory reactions, extending to the submucosa. These results were consistent with Gazzinelli-Guimaraes and Nutman [67], and Sorobetea et al. [68]. Furthermore, we reported mechanical damage in both intestinal and skeletal muscle cells with the accumulation of inflammatory cells. This could be due to the direct mechanical action of the parasite, causing pathological reactions. In the intestine, the administration of pumpkin seed oil to the infected mice restored the normal architecture of the intestinal tissue and reduced the inflammatory reaction. On the other hand, we noted the increase in the number of goblet cells in all infected groups which are usually associated with the invasion of *T. spiralis* [69, 70].

Bruschi and Chiumiento [71] reported that the inflammation of skeletal muscles correlated with high levels of oxygen reactive species and other free radicals. Interestingly, we reported a statistically significant decrease in the mean number of encysted larvae in the diaphragm and muscle at 54 dpi, suggesting that pumpkin seed oil contains active metabolites that might have a nematocidal effect. Additionally, it might be due to the degenerative effect of pumpkin seed oil on the reproductive organs of the parasites [72] and control of parasite fertility [73]. The anthelminthic effect of pumpkin seed extract in vivo studies was dependent on time and dose [17]. Although there was a non-significant difference between the efficiency of pumpkin seed oil in the present study compared to in vitro studies. This might be due to the effect of gastrointestinal factors such as PH that may change the response of parasite worms to treatment [74].

The anti-inflammatory effects of pumpkin seed oil could be attributed to the putative long-term health effects of isocaloric switch by partial replacement of saturated fats by unsaturated fats [75]. MMP-9 is recommended in the recent literature as an indirect marker to assess myositis severity in infected hosts [32, 76]. Our results clearly demonstrated a highly intracellular staining of MMP-9 in mononuclear cells infiltrating the lamina propria in the intestine and muscle tissues starting one week post infection in the infected-untreated group. This could be attributed cell-mediated reaction of macrophages that can induce large amounts of MMP production [77]. This was correlated with the fibrotic process in the intestinal and muscular diseases, which agreed with the results of Von Lampe et al. [78]. To the best of our knowledge, little is known about the effectiveness of pumpkin oil in the activation of MMP-9 during infection. The PI3K/Akt/NF-κB signaling pathway is indeed well-documented as a crucial regulator of MMP9 expression, particularly in inflammatory processes. Studies have shown that the activation of PI3K/Akt leads to the activation of NF-κB, which in turn upregulates MMP9 transcription [79, 80]. This pathway plays a critical role in mediating the inflammatory response, contributing to various pathological conditions, including those influenced by dietary components such as oils. For instance, omega-3 fatty acids, commonly found in fish oil, have been shown to inhibit this pathway, thereby reducing MMP9 expression and attenuating inflammation [81]. In our study primarily, we focus on the direct measurement of MMP9 as an inflammatory mediator in response to pumpkin oil treatments against *T. spiralis* infection. As pumpkin seed oil rich with carotenoids, phenolic compounds, and flavonoids, it modulates COX-2 gene which contain consensus sequences for NF-κB and play key role in inflammatory process. Pro-inflammatory stimuli activate the complex containing the NF-κB essential modulator (NEMO) and IκB kinase (IKK)1/2. IKK1/2 phosphorylates IκBs by IKK signalosome complex [82]. In this study, the pumpkin oil showed a statistically significant reduction in the expression of MMP-9 in the infected mice, suggesting its impact as anti-inflammatory agents and stimulation immune responses. The obtained results are promising for the putative anti-inflammatory effect of pumpkin seeds and an integrated approach to control inflammatory diseases.

## Conclusion

Considering the outcomes of our results, pumpkin seed oil could be used as an effective alternative drug, characterized by costless and high clinical safety. Our results reported the therapeutic impact of pumpkin seed oil against parasitic infection and inflammatory diseases. More investigations are needed to elucidate the active components of *Cucurbita* species responsible for their anti-inflammatory agent.

## Supplementary Information

Supplementary Material 1

## Data Availability

No/Not applicable (this manuscript does not report data generation or analysis).

## Data availability

No datasets were generated or analysed during the current study.
