# Supplementary figures and images for: Pumpkin seed oil: unveiling its potential in controlling inflammation and pathogenicity during experimental trichinellosis
Source: BMC Vet Res. 2024 Sep 20;20:419. doi: 10.1186/s12917-024-04241-2 (PMC11414094; doi:10.1186/s12917-024-04241-2)

Supplementary file (1): Transverse section of a mice tonsil as a confirmed control of MMP-9 marker

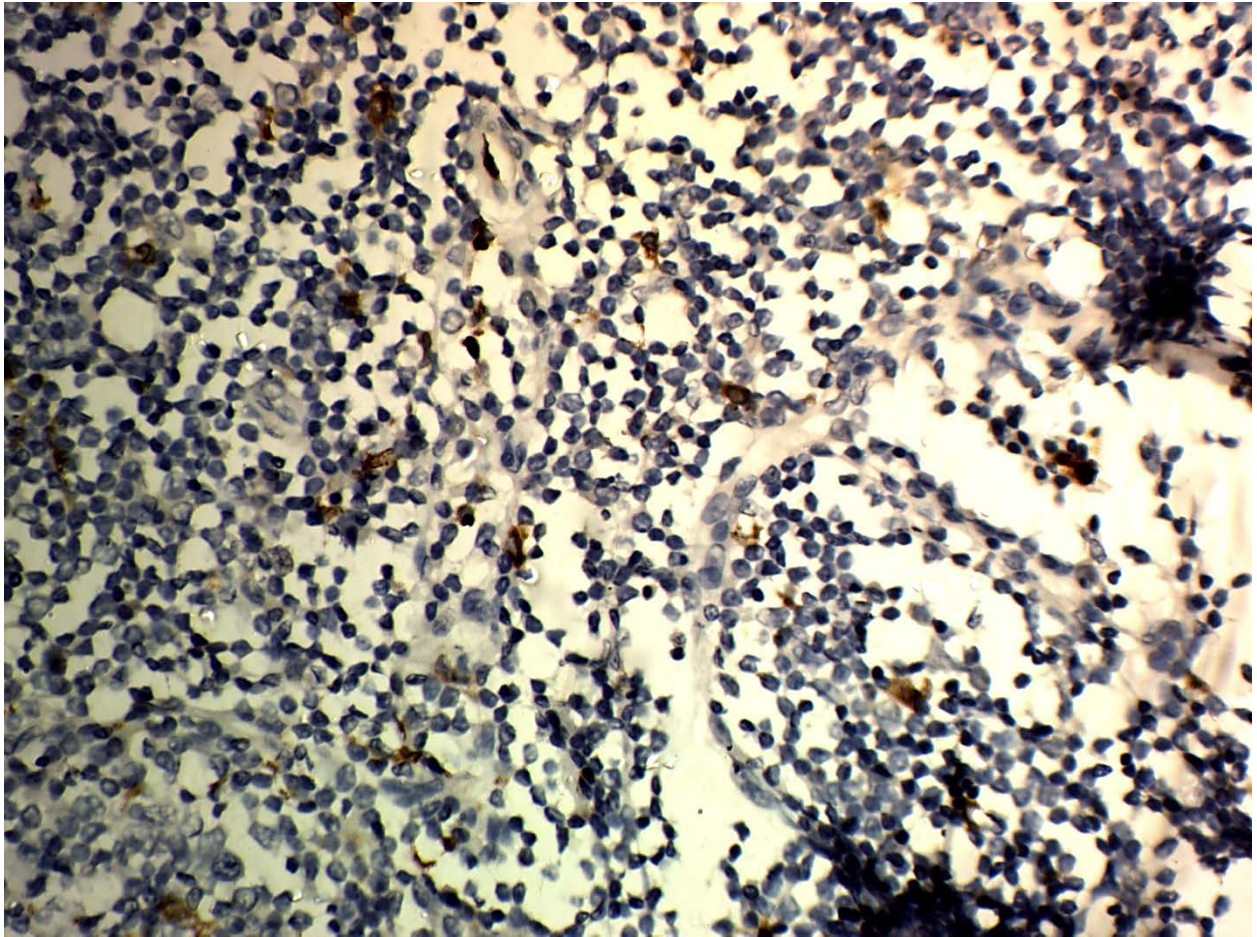

Supplement: Supplementary file 1 — Supplementary Material 1 [file 12917_2024_4241_MOESM1_ESM.pdf]
